# The Relationship Between the Effects of Lateral Wedge Insoles and Kinematic Chain Dynamics Between the Hindfoot and Lower Leg in Patients With Osteoarthritis of the Knee

**DOI:** 10.7759/cureus.37624

**Published:** 2023-04-15

**Authors:** Masahiro Edo, Gaku Nishizawa, Yuto Matsumura, Nobuhiro Nemoto, Naoki Yotsumoto, Shin Kojima

**Affiliations:** 1 Department of Rehabilitation Sciences, Chiba Prefectural University of Health Sciences, Chiba, JPN; 2 Department of Rehabilitation, Medical Plaza Ichikawa Station, Chiba, JPN; 3 Department of Rehabilitation, Itabashi Medical System (IMS) Tokyo Katsushika General Hospital, Tokyo, JPN; 4 Department of Orthopedic Surgery, Itabashi Medical System (IMS) Tokyo Katsushika General Hospital, Tokyo, JPN

**Keywords:** inertial measurement unit, gait, kinematic chain, lateral wedge insole, lateral thrust, knee osteoarthritis

## Abstract

Purpose

We aim to determine whether kinematic chain dynamics of the hindfoot and lower leg are involved in the effect of a lateral wedge insole (LWI) on reducing lateral thrust among patients with medial compartment knee osteoarthritis (KOA).

Participants and methods

Eight patients with knee osteoarthritis were included in the study. Evaluation of the kinematic chain and gait analysis was performed using an inertial measurement unit (IMU). The dynamics of the kinematic chain were calculated as linear regression coefficients of the external rotation angle of the lower leg relative to the inversion angle of the hindfoot during repeated inversion and eversion of the foot in the standing position (kinematic chain ratio (KCR)). Walk tests were performed under four conditions: barefoot (BF), neutral insole (NI) with an incline of 0 degrees, and LWI with an incline of approximately 5 and 10 degrees (5LWI and 10LWI, respectively).

Results

The mean (± standard deviation (SD)) KCR was 1.4 ± 0.5. The KCR was significantly correlated with the change in 5LWI lateral thrust acceleration relative to BF (r = 0.74). A significant correlation was also observed between changes in the hindfoot evolution angle and lower leg internal rotation angle with a 10LWI with respect to BF and NI, and changes in lateral thrust acceleration.

Conclusion

The results of this study suggest that the kinematic chain is involved in the effects of an LWI in patients with knee osteoarthritis.

## Introduction

Osteoarthritis of the knee is a chronic progressive disease that causes pain, knee stiffness, instability, functional deterioration, and other effects due to damage to articular cartilage and surrounding tissues. One cause of these pathological changes is thought to be the accumulation of biomechanical imbalances [1]. The external knee adduction moment (KAM) is considered to be a surrogate indicator of compressive stress in the medial compartment of the knee joint, which is known to be involved in the progression of medial compartment knee osteoarthritis (KOA) [2]. A lateral wedge insole (LWI) has been shown to reduce KAM by shortening the lever arm of the floor reaction force at the knee joint by moving the center of pressure outward [3]. However, while many studies have reported that an LWI reduces KAM [3-6], there are scattered reports of no reduction [7,8]; as such, there is a need to clarify the characteristics of patients who respond positively to LWI [9,10].

Regarding such patient characteristics, it has been reported that normal foot alignment and large hindfoot eversion during the early stance phase are associated with the susceptibility of an LWI in reducing KAM [11,12]. In addition to these characteristics, we believe that kinematic chain dynamics between the hindfoot and the lower leg may also be involved. The kinematic chain is an engineering concept that regards the body as a link mechanism in which multiple body segments are connected and indicates that the movement of one joint affects that of another joint [13]. For example, it is known that in situations in which the foot is loaded, such as walking, the frontal plane motion of the hindfoot (i.e., inversion/eversion) and the horizontal plane motion of the lower leg (external/inner rotation) are closely linked [14]. However, the ratio of this linkage has been found to vary greatly among individuals [15], and individual differences in this dynamic may mediate the effect of an LWI in reducing KAM.

As previously mentioned, KAM is an important index in the progression of knee osteoarthritis [2]; however, its measurement requires expensive equipment and a dedicated experimental space; thus, its measurement in clinical settings is quite difficult [16]. Therefore, lateral thrust is attracting attention as an alternative indicator of KAM. Lateral thrust, similar to KAM, contributes to compressive stress on the medial compartment of the knee joint and may be a more acute indicator than KAM because it can be recognized in the early stages of KOA [17,18]. Moreover, the acceleration of lateral thrust, measured much more simply than KAM using an inertial measurement unit (IMU), has also been confirmed to be correlated with KAM [19].

Therefore, the purpose of this study was to clarify whether kinematic chain dynamics of the hindfoot and lower leg are involved in the suppression of lateral thrust by the use of an LWI among patients with KOA. Specifically, we analyzed the correlation between lateral thrust acceleration during walking and the ratio of hindfoot inversion/eversion and lower leg rotation generated as part of the kinematic chain.

## Materials and methods

The present study was conducted using a within-subject design and included eight patients diagnosed with KOA by an orthopedic surgeon. The timeframe for data collection was from August 2018 to January 2019. Participants consisted of one male and seven females, with a mean (± standard deviation (SD)) age of 71.0 ± 6.4 years, a height of 155.6 ± 9.6 cm, and a weight of 66.5 ± 8.8 kg. Among the limbs measured, three were grade II and five were grade III according to the Kellgren-Lawrence grade. In the case of bilateral KOA, the more severely affected side was considered to be the side with KOA. The study was approved by the Ethics Review Committee of Bunkyo Gakuin University (approval number 2017-0047), and participants provided informed written consent to participate.

**Figure 1:**
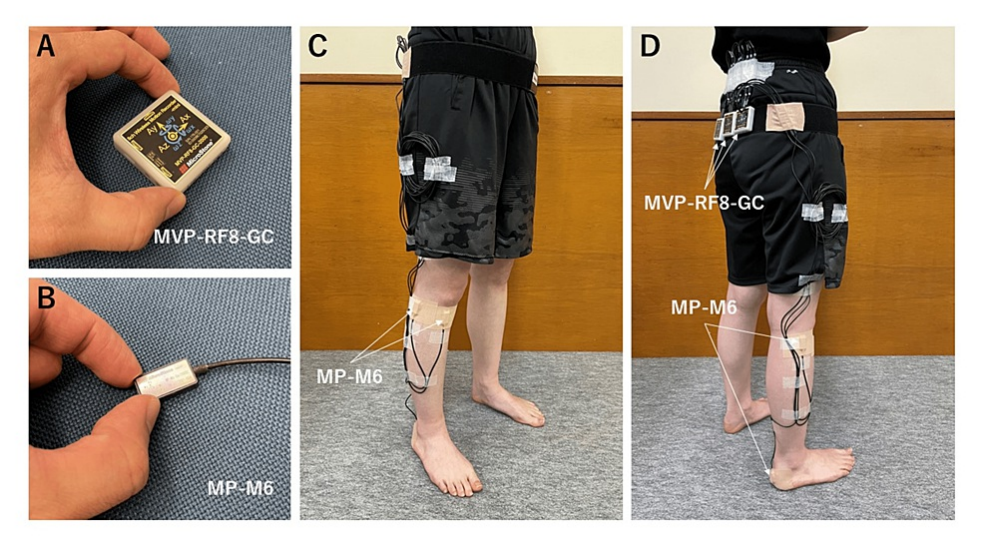
Appearance and installation method of the IMUs The appearance (A and B) and installation method (C and D) of the IMUs used in this study are shown. IMUs: inertial measurement units

IMUs (three MP-M6 and three MVP-RF8-GC, Microstone, Nagano, Japan; all at 200 Hz) were used to perform measurements (Figure 1). The MP-M6 is a small, lightweight wired sensor measuring 23 × 12 × 5 mm and weighing 10 g. Since it can be brought into close contact with the body surface, it can accurately measure body movements. The MVP-RF8-GC is a wireless sensor measuring 45 × 45 × 12 mm and weighing 25 g. It can also be used as an IMU itself or as a transmitter for importing data measured by MP-M6 into a personal computer (PC) through a wired connection. The MP-M6 was affixed to the posterior surface of the heel, medial aspect of the tibial ridge, and fibular head of the measured limb using double-sided tape and then fixed over the MP-M6 with elastic tape to prevent the effects of soft tissue vibration. The MVP-RF8-GCs were fixed to the waist using a belt and used to import measurement data from the MP-M6, which was externally inputted via a wired connection, to a personal computer (PC) via “Bluetooth.”

**Figure 2:**
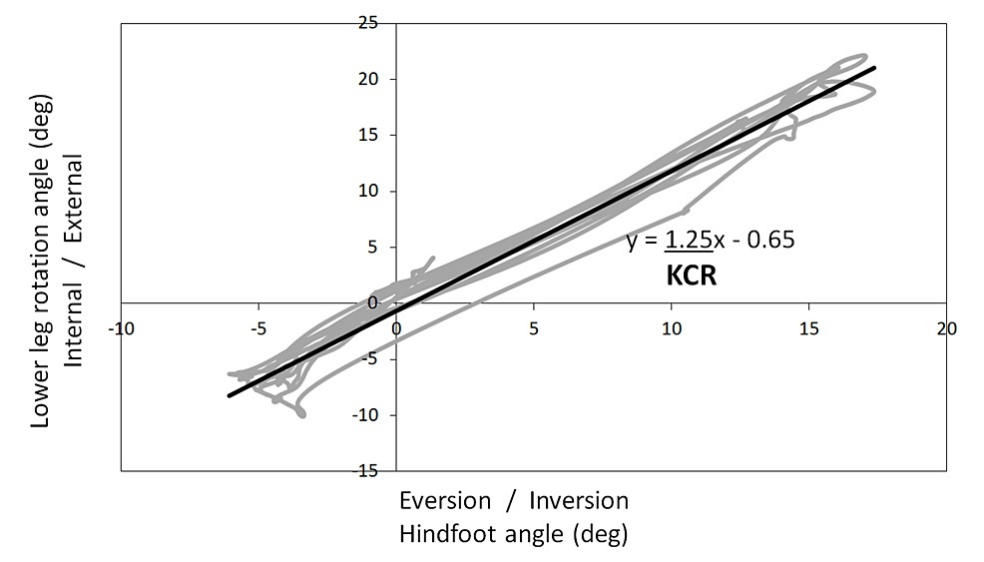
Definition of KCR (representative example) KCR: kinematic chain ratio

The movements measured were those used to evaluate kinematic chain dynamics of the foot and gait, both of which were performed in a standing stationary position at the beginning and end of the movement. The movements used to evaluate kinematic chain dynamics included five active repetitions of inversion and eversion of both feet in the standing position, based on the authors’ previous study [15]. The lower leg rotation angle relative to the hindfoot inversion and eversion angles during this movement has been found to fit the first-order regression equation well, and the first-order regression coefficient was defined as the kinematic chain ratio (KCR) as an index of kinematic chain dynamics (Figure 2) [15]. A KCR of >1.0 represents a kinematic chain that causes a large amount of leg rotation, whereas a KCR of <1.0 represents a kinematic chain that causes a large amount of hindfoot inversion/eversion. Gait was performed by each participant on a 15 m path at a comfortable speed under four conditions: barefoot (BF), neutral insole (NI) with a height of 10 mm and an incline of 0 degrees, and LWIs with an incline of approximately 5 degrees (5LWI) and 10 degrees (10LWI). The NIs and LWIs were fabricated from silicone rubber and belted to the foot (Nakamura Brace, Shimane, Japan) on both sides; no shoes were worn. The tilt angle of the LWI was determined based on the report [20] that KAM decreased at 5 and 10 degrees. NI was the condition in which only the influence of inclination was excluded. The insoles were available in three sizes, and each participant wore the one that best fits them. The same products are widely used in Japan and have previously been reported [11,21,22]. Each condition was performed in random order, and participants were permitted five minutes of practice to acclimate to each condition before measurements were performed.

Regarding data processing, angular velocity data acquired by the IMU on the posterior heel and medial aspect of the tibial ridge as read by the PC were integrated. The slope of the baseline due to drift accumulated over time by integrating the angular velocity was estimated from the values at the beginning and end of the movement using a regression line. Thereafter, the slope was subtracted and corrected to obtain the hindfoot eversion angle and lower leg internal rotation angle [23]. The KCR was then calculated for kinematic chain dynamics. For gait, the timing of heel contact was determined from the spike waveform of the IMU acceleration data on the posterior heel, and the amount of change in the hindfoot eversion angle and the amount of change in the lower leg internal rotation angle were determined using 0%-12% of the gait cycle [24], corresponding to the initial contact to the loading response as the analysis interval. Since the lateral thrust also occurs early in the stance phase [25], the peak value of the lateral acceleration of the IMU at the fibular head during this period was defined as lateral thrust acceleration. For analysis, gait data were averaged over eight steps.

For statistical analysis, the data normality of all parameters was examined using the Shapiro-Wilk test. Differences in gait parameters in the four insole conditions were then examined using a one-way repeated-measures analysis of variance (ANOVA). In addition, the relationship between the change in each gait parameter across conditions and KCR was examined using Pearson’s product-rate correlation coefficient. Statistical analysis was performed using Statistical Package for the Social Sciences (SPSS) version 27 (IBM Corporation, Armonk, NY, USA), and the significance level for all tests was 5%.

## Results

The mean (± SD) KCR was 1.4 ± 0.5. The lateral thrust acceleration, the amount of change in the hindfoot eversion angle, and the amount of change in the lower leg internal rotation angle under each insole condition are summarized in Table 1. No significant differences were found in any of the items. The correlations between lateral thrust acceleration and changes in lower limb motion and KCR between each condition are summarized in Table 2. The greater the KCR (i.e., kinematic chain with greater lower leg rotation), the more lateral thrust acceleration decreased at 5LWI relative to BF. In addition, the greater the hindfoot eversion and lower leg internal rotation motion that occurred at 10LWI than at BF and NI, the more lateral thrust acceleration decreased.

**Table 1:** Gait parameters among different insole conditions One-way repeated-measures ANOVA: all n.s. ANOVA: analysis of variance, BF: barefoot, NI: neutral insole, 5LWI: lateral wedge insole with an incline of 5 degrees, 10LWI: lateral wedge insole with an incline of 10 degrees, LTA: lateral thrust acceleration

		BF	NI	5LWI	10LWI
LTA	(m/s^2^)	18.0 ± 3.7	19.5 ± 4.8	19.1 ± 4.4	17.2 ± 2.4
Amount of change in the hindfoot eversion angle	(degree)	2.8 ± 1.7	2.2 ± 2.2	2.8 ± 2.0	4.5 ± 1.9
Amount of change in the lower leg internal rotation angle	(degree)	1.8 ± 3.3	4.0 ± 2.9	4.7 ± 2.1	1.3 ± 2.6

**Table 2:** Correlation coefficients for change in lateral thrust and lower limb motion for each insole condition Pearson’s product-moment correlation coefficient: *p < 0.05 Except for KCR, the analysis determined the amount of change between each condition, e.g., ΔNI–BF reflects the amount of change in NI relative to BF. KCR: kinematic chain ratio, BF: barefoot, NI: neutral insole, 5LWI: lateral wedge insole with an incline of 5 degrees, 10LWI: lateral wedge insole with an incline of 10 degrees

		KCR	Amount of change in the hindfoot eversion angle (degree)	Amount of change in the lower leg internal rotation angle (degree)
Lateral thrust acceleration (m/s^2^)	ΔNI-BF	0.42	0.34	-0.48
	Δ5LWI-BF	0.74*	-0.26	-0.43
	Δ10LWI-BF	0.08	-0.83*	0.04
	Δ5LWI-NI	0.55	-0.12	-0.41
	Δ10LWI-NI	-0.19	-0.56	-0.86*

## Discussion

This study was performed to clarify whether kinematic chain dynamics of the hindfoot and lower leg are involved in the effect of an LWI on reducing lateral thrust acceleration in patients with KOA. The results of this study partially support the hypothesis that the greater the KCR and the greater the hindfoot eversion and lower leg internal rotation produced by an LWI, the greater the reduction in lateral thrust acceleration.

The fact that there was no significant difference in lateral thrust acceleration in each insole condition was consistent with previous studies in that the effect of an LWI differs among individuals [7,8], suggesting that kinematic chain dynamics, in addition to previously reported foot alignment and foot motion [11,12], are involved in the background. More specifically, the greater the KCR (kinematic chain with greater lower leg rotation), the more the LWI may suppress lateral thrust, or the greater the hindfoot eversion and lower leg internal rotation produced by the LWI, the more the LWI may suppress lateral thrust. During the loading response phase of normal gait, the hindfoot moves from a mildly inverted position to an everted one, which in turn causes internal rotation of the lower leg by kinematic chain action [24]. This lower leg internal rotation is believed to contribute to the suppression of external leg tilt, although it has been reported that patients with KOA exhibit smaller internal rotation of the lower leg [26]. These findings suggest that there may be a mechanism by which an LWI causes eversion of the hindfoot, which leads to internal rotation of the lower leg, which in turn suppresses lateral thrust. In this mechanism, if the KCR was small, the internal rotation of the lower leg was small even if the LWI caused the eversion of the hindfoot in the early stage of stance. As a result, it is inferred that the lateral thrust is also not suppressed. Conversely, if the KCR is large, it is inferred that the hindfoot eversion by the LWI is efficiently converted into internal rotation of the lower leg, suppressing the lateral thrust. Further studies are needed to verify this mechanism; however, if this is the case, it is anticipated to enable screening of patients with indications for an LWI.

The KCR of patients with KOA measured in this study was generally equivalent to that reported in a previous study using a three-dimensional motion analyzer in patients with nearly equivalent conditions (1.5 ± 0.6) [27]. Although not a direct validation, the results suggest the possibility that an IMU, which is less expensive and has fewer restrictions overall, can be used to evaluate KCR with the same accuracy as a three-dimensional motion analyzer. In other words, KCR could be evaluated along with lateral thrust in clinical practice [19]. This secondary fact may be considered the strength of this research.

The present study, however, had several limitations. First, the sample size was small for the results to be generalized. In addition, the relationship between each parameter was examined in terms of a single correlation. Because the kinematic chain represents a linkage of multiple body segments, multivariate analysis is desirable, and this would have required an increased number of participants. Because the hindfoot and lower leg angles used in this study were calculated by integrating angular velocities and were relative to the start of the movement, this study primarily addressed changes in these angles, not the angles themselves. It cannot be ruled out that factors such as gait speed, which affect lateral thrust, may have varied with each insole condition. Long-term effects are also unknown. Although it has several areas for improvement in terms of the lateral thrust inhibition effect caused by LWI, in addition to the previously reported factor of the severity of knee osteoarthritis [22], this study demonstrates the possibility that the dynamics of the kinematic chain are also involved.

## Conclusions

In conclusion, the findings of the present study suggest that in KOA patients using an LWI, the greater the lower leg rotation in the kinematic chain or the more a linkage between hindfoot eversion and lower leg internal rotation during the loading response phase, the greater the decrease in lateral thrust. In other words, this study suggests that the kinematic chain dynamics of the hindfoot and lower leg are involved in the suppression of lateral thrust by the use of an LWI among patients with KOA. Although there is a need for further research, the determination of KOA patients who respond positively to LWI may need to focus on subjects with kinetic chain characteristics of greater lower leg rotation.
